# Cerebellar c9RAN proteins associate with clinical and neuropathological characteristics of *C9ORF72* repeat expansion carriers

**DOI:** 10.1007/s00401-015-1474-4

**Published:** 2015-09-08

**Authors:** Tania F. Gendron, Marka van Blitterswijk, Kevin F. Bieniek, Lillian M. Daughrity, Jie Jiang, Beth K. Rush, Otto Pedraza, John A. Lucas, Melissa E. Murray, Pamela Desaro, Amelia Robertson, Karen Overstreet, Colleen S. Thomas, Julia E. Crook, Monica Castanedes-Casey, Linda Rousseau, Keith A. Josephs, Joseph E. Parisi, David S. Knopman, Ronald C. Petersen, Bradley F. Boeve, Neill R. Graff-Radford, Rosa Rademakers, Clotilde Lagier-Tourenne, Dieter Edbauer, Don W. Cleveland, Dennis W. Dickson, Leonard Petrucelli, Kevin B. Boylan

**Affiliations:** Department of Neuroscience, Mayo Clinic, Jacksonville, FL 32224 USA; Mayo Graduate School, Mayo Clinic, Rochester, MN 55905 USA; Ludwig Institute, University of California at San Diego, La Jolla, CA 92093 USA; Department of Psychiatry and Psychology, Mayo Clinic, Jacksonville, FL 32224 USA; Department of Neurology, Mayo Clinic, Jacksonville, FL 32224 USA; Section of Biostatistics, Mayo Clinic, Jacksonville, FL 32224 USA; Department of Neurology, Mayo Clinic, Rochester, MN 55905 USA; Department of Neurosciences, University of California at San Diego, La Jolla, CA 92093 USA; German Center for Neurodegenerative Diseases (DZNE), Munich, Germany; Institute for Metabolic Biochemistry, Ludwig-Maximilians University Munich, Munich, Germany; Munich Cluster of Systems Neurology (SyNergy), Munich, Germany; Department of Cellular and Molecular Medicine, University of California at San Diego, La Jolla, CA 92093 USA

**Keywords:** Amyotrophic lateral sclerosis, *C9ORF72* repeat expansion, c9RAN proteins, Cognition, Dipeptide repeat proteins, Frontotemporal dementia, Frontotemporal lobar degeneration, Neuropathological diagnosis, Repeat-associated non-ATG translation

## Abstract

Clinical and neuropathological characteristics associated with G_4_C_2_ repeat expansions in chromosome 9 open reading frame 72 (*C9ORF72*), the most common genetic cause of amyotrophic lateral sclerosis (ALS) and frontotemporal dementia, are highly variable. To gain insight on the molecular basis for the heterogeneity among *C9ORF72* mutation carriers, we evaluated associations between features of disease and levels of two abundantly expressed “c9RAN proteins” produced by repeat-associated non-ATG (RAN) translation of the expanded repeat. For these studies, we took a departure from traditional immunohistochemical approaches and instead employed immunoassays to quantitatively measure poly(GP) and poly(GA) levels in cerebellum, frontal cortex, motor cortex, and/or hippocampus from 55 *C9ORF72* mutation carriers [12 patients with ALS, 24 with frontotemporal lobar degeneration (FTLD) and 19 with FTLD with motor neuron disease (FTLD-MND)]. We additionally investigated associations between levels of poly(GP) or poly(GA) and cognitive impairment in 15 *C9ORF72* ALS patients for whom neuropsychological data were available. Among the neuroanatomical regions investigated, poly(GP) levels were highest in the cerebellum. In this same region, associations between poly(GP) and both neuropathological and clinical features were detected. Specifically, cerebellar poly(GP) levels were significantly lower in patients with ALS compared to patients with FTLD or FTLD-MND. Furthermore, cerebellar poly(GP) associated with cognitive score in our cohort of 15 patients. In the cerebellum, poly(GA) levels similarly trended lower in the ALS subgroup compared to FTLD or FTLD-MND subgroups, but no association between cerebellar poly(GA) and cognitive score was detected. Both cerebellar poly(GP) and poly(GA) associated with *C9ORF72* variant 3 mRNA expression, but not variant 1 expression, repeat size, disease onset, or survival after onset. Overall, these data indicate that cerebellar abnormalities, as evidenced by poly(GP) accumulation, associate with neuropathological and clinical phenotypes, in particular cognitive impairment, of *C9ORF72* mutation carriers.

**Electronic supplementary material:**

The online version of this article (doi:10.1007/s00401-015-1474-4) contains supplementary material, which is available to authorized users.

## Introduction

Amyotrophic lateral sclerosis (ALS), the most common motor neuron disease (MND), is characterized by progressive upper and lower motor neuron weakness, typically resulting in death from ventilatory failure within three to five years of onset. In addition, up to 50 % of ALS patients develop cognitive impairments consistent with frontotemporal dementia (FTD) [15]. FTD, the clinical presentation of frontotemporal lobar degeneration (FTLD), encompasses a group of disorders characterized by changes in personality, behavior, and/or language. A proportion of FTD patients also develop ALS [33]. The clinical overlap between ALS and FTD, combined with neuropathological similarities—TDP-43 pathology is present in the majority of ALS cases and the most common pathological subtype of FTLD (FTLD-TDP)—suggest ALS and FTD are part of a disease spectrum. This concept was strengthened by the discovery that a G_4_C_2_ repeat expansion in chromosome 9 open reading frame 72 (*C9ORF72*) is the most common genetic cause of ALS and FTD [10, 28]. The molecular basis for the clinical variation among *C9ORF72* patients is not known. In addition to TDP-43 pathology, patients with “c9FTD/ALS” develop cerebellar and hippocampal TDP-43-negative neuronal inclusions that are positive for p62, ubiquitin, and select ubiquitin-binding proteins [1, 5, 6, 23, 25]. Other hallmark features of c9FTD/ALS result from the accumulation of sense G_4_C_2_ or antisense G_2_C_4_ repeat-containing RNA. These transcripts form nuclear RNA foci in cells throughout the central nervous system and are subject to repeat-associated non-ATG (RAN) translation [3, 10, 11, 21, 22, 43]. RAN translation of sense and antisense repeats, which occurs in all reading frames in the absence of an initiating ATG codon, yields five proteins of repeating dipeptides (GP, GA, GR, PR, PA), collectively referred to as c9RAN proteins. c9RAN proteins are a major component of TDP-43-negative, p62-positive inclusions [18]. Indeed, c9RAN protein pathology, in particular inclusions of poly(GP) or poly(GA), is abundant in cerebellum, hippocampus, and neocortical regions [3, 16, 18, 21, 22, 30, 43].

Several c9RAN proteins are toxic in a variety of models, thus implicating RAN translation as a mechanism of disease [12, 14, 19, 20, 35, 37, 40–43]. However, there have been conflicting reports on clinical and neuropathological correlations with c9RAN protein pathology in *C9ORF72* mutation carriers [9, 16, 18, 30]. For instance, among three studies, no associations between poly(GA) inclusion burden in various brain regions and either neurodegeneration, clinical phenotype, or neuropathological phenotype were detected [9, 16, 18]. Conversely, it was recently reported that, in a cohort of 14 *C9ORF72* patients, poly(GA) inclusions in cerebellar granule cells were significantly more abundant in patients with FTLD compared to patients with MND or FTLD-MND [30]. These findings highlight the need for more thorough evaluations of the various c9RAN proteins in c9FTD/ALS, and that neuroanatomical regions not typically studied, such as the cerebellum, should be included in these investigations. Indeed, while often overlooked, neuroimaging and clinical evidence support the involvement of the cerebellum in cognitive and affective regulation [13]. Combined with cerebellar atrophy emerging as a distinguishing feature of c9FTD/ALS compared to sporadic or other familial forms of these diseases [17, 32, 38], and our discovery of robust cerebellar transcriptome changes in c9ALS [27], the question arises as to whether cerebellar pathology contributes to cognitive dysfunction in *C9ORF72* mutation carriers. We thus evaluated whether poly(GP) and poly(GA), which are abundantly expressed in the cerebellum, associate with neuropathological and clinical characteristics of patients with a *C9ORF72* repeat expansion.

## Materials and methods

### Study design and participants

To evaluate associations between c9RAN protein levels and neuropathological diagnosis, we included all cases in the Mayo Clinic Jacksonville brain bank who were identified as *C9ORF72* expansion carriers by repeat-primed PCR [10] and by immunostaining for poly(GP) pathology [3], and for whom frozen tissue from the cerebellum, frontal cortex, motor cortex, and hippocampus was available (*n* = 55; Table 1). Family history of disease was considered positive if patients were known to have a first or second degree relative with ALS, FTLD or mixed features of both. Age at onset was estimated based on the appearance of first disease symptoms.

**Table 1:** Characteristics of *C9ORF72* repeat expansion carriers

	Overall cohort (*n* = 55)	ALS (*n* = 12)	FTLD (*n* = 24)	FTLD-MND (*n* = 19)
Women	30 (55 %)	9 (75 %)	8 (33 %)	8 (42 %)
Age of onset (years)	61.6 (52.8–67.1)	51.0 (47.8–65.5)	66.2 (56.3–70.0)	57.5 (54.2–65.0)
Age of death (years)	66.5 (59.1–72.6)	56.0 (49.9–67.1)	72.4 (66.2–80.6)	62.0 (59.9–68.1)
Survival after onset (years)	5.0 (2.7–8.0)	2.4 (1.5–3.2)	7.3 (5.7–9.8)	4.2 (2.6–6.7)
Family history	22 (47 %)	4 (36 %)	9 (45 %)	9 (56 %)

Data presented as median (IQR) or number (%)

For some patients, information was unavailable for age of onset and survival after onset (*n* = 3, 2 with FTLD and 1 with FTLD-MND) and family history (*n* = 8, 1 with ALS, 4 with FTLD and 3 with FTLD-MND)

*ALS* amyotrophic lateral sclerosis, *FTLD* frontotemporal lobar degeneration, *FTLD-MND* frontotemporal lobar degeneration with motor neuron disease

Of the 55 patients in our overall cohort, 15 composed our clinical cohort. An ALS-focused selection established a *C9ORF72* patient cohort in which cognitive status ranged from normal to definitely abnormal. Study patients in the clinical cohort were ascertained from 2009 to 2014 on the basis of a diagnosis of ALS meeting El Escorial criteria, participation in the Mayo Clinic ALS Center deeded autopsy program, and genetically and pathologically confirmed to have a *C9ORF72* repeat expansion (*n* = 15; Table 4; Supplementary Table 1). All patients in the clinical cohort were followed for multiple visits by the same neurologist (KBB) from initial encounter through advanced disease stage with severe, global disability. Assessments of cognitive function were derived from independent review of clinical records and test results by four evaluators, a neurologist (KBB) and three neuropsychologists (JAL, OP, and BKR), who all had access to the same clinical data. Clinical records for all patients included observations by clinical staff and family members involved in the patients’ care with regard to cognition and behavior, and for nine of the 15 patients, data from the ALS Cognitive Behavioral Screen, a validated neuropsychological screening instrument [39], and/or a full neuropsychological examination (Supplementary Table 1). Examiners were aware of the patient’s diagnosis of c9ALS but were blinded to data on c9RAN protein levels. Owing to varied data available across the cohort on patients’ neuropsychological status, each patient with ALS was independently assessed by each of the evaluators on a 0–2 scale (0 = normal, 1 = mild or questionable impairment, or 2 = definitely abnormal) with respect to cognition. Scores were based on assessment of executive function, verbal fluency, and memory as supported by observational and quantitative data. Given our comparisons of cognitive status with autopsy findings, the assigned scores reflected the evaluator’s assessments of the patient’s cognitive function in late stage disease (i.e. near death with severe, global disability) based on available supporting data, which in all cases included caregiver information regarding the patient’s cognitive status at an advanced stage of disease. The rating of the 15 patients by the four evaluators resulted in 90 potential pairwise agreements; pairwise agreement for the cognitive score was 80 % (72/90). The final cognitive score for each patient represents the average of the scores of the four evaluators.

Written informed consent was obtained from all participants or their legal next of kin if they were unable to give written consent, and biological samples were obtained with ethics committee approval.

### Immunoassay analysis of c9RAN proteins in tissue homogenates

Brain homogenates were prepared as previously described [2]. In brief, approximately 50 mg of cerebellum, frontal cortex, motor cortex, or hippocampus tissue was homogenized in cold RIPA buffer (25 mM Tris–HCl pH 7.6, 150 mM NaCl, 1 % sodium deoxycholate, 1 % Nonidet P-40, 0.1 % sodium dodecyl sulfate, protease and phosphatase inhibitors) and sonicated on ice. Homogenates were cleared by centrifugation at 100,000*g* for 30 min at 4 °C. The supernatant was collected and the concentration of RIPA-soluble proteins was determined by BCA assay. The resulting pellet was resuspended in RIPA buffer and, to prevent carry-over, re-sonicated and re-centrifuged. The RIPA-insoluble pellet was then extracted using 7 M urea buffer, sonicated and centrifuged at 100,000*g* for 30 min at 22 °C. The protein concentration of the urea-soluble supernatant was determined by Bradford assay.

Poly(GP) levels in lysates were measured using a previously described sandwich immunoassay that utilizes an affinity purified rabbit polyclonal poly(GP) antibody (Rb9259) and Meso Scale Discovery (MSD) electrochemiluminescence detection technology [34]. This assay specifically detects poly(GP) and no other c9RAN protein [34]. Homogenates were diluted in Tris-buffered saline (TBS) and equal amounts of protein for all samples from the same neuroanatomical region and fraction were tested in duplicate wells. Serial dilutions of recombinant (GP)_8_ in TBS were used to prepare the standard curve. Response values corresponding to the intensity of emitted light upon electrochemical stimulation of the assay plate using the MSD QUICKPLEX SQ120 were acquired and background corrected using the average response from homogenates obtained from non-*C9ORF72* repeat expansion carriers prior to interpolation of poly(GP) levels [presented as ng of poly(GP) per mg protein] using the standard curve. Poly(GA) levels in homogenates were similarly measured with an MSD-based sandwich immunoassay that utilizes a purified rabbit polyclonal poly(GA) antibody (Rb4333) provided by the Cleveland lab and a mouse monoclonal poly(GA) antibody (clone 5F2, [16]) provided by the Edbauer lab. Serial dilutions of recombinant (GA)_50_ were used for the standard curve. This assay specifically detects poly(GA) and no other c9RAN protein (Supplementary Fig. 1).

### Immunohistochemistry and analysis of poly(GP) and phosphorylated TDP-43 pathology

To compare poly(GP) levels evaluated by immunoassay to poly(GP) pathology measured by immunohistochemistry, cerebellar tissue from 35 *C9ORF72* repeat expansion carriers were immunostained for poly(GP). In addition, phosphorylated TDP-43 (pTDP-43) pathology was evaluated in frontal cortex and hippocampus tissues from the 15 patients in our clinical cohort to study associations of pTDP-43 with cognitive score or neuropathological diagnosis. Five micron thick slices were cut from formalin-fixed, paraffin-embedded tissue, and mounted on glass slides. After drying, slides were deparaffinized and rehydrated in xylene and alcohol washes before being steamed for 30 min in deionized water for antigen retrieval. All slides were processed on a Dako Autostainer with the Dako EnVision™+ system and 3′3-diaminobenzidine chromogen. Slides were stained with rabbit polyclonal poly(GP) antiserum (Rb5823, 1:5000) [3], rabbit polyclonal poly(GA) antiserum (Rb9880, 1:30,000) [42], or a mouse monoclonal antibody against TDP-43 phosphorylated at S409/S410 (1:5000, Cosmo Bio Co., Tokyo, Japan), then counterstained with Lerner hematoxylin and coverslipped with Cytoseal permanent mounting media.

To quantify poly(GP) pathology in the cerebellum, high resolution images of immunostained slides were captured using an Aperio ScanScope XT scanner and analyzed using ImageScope software (Leica Biosystems, Buffalo Grove, IL). For each case, twenty 1 mm^2^ annotated boxes were placed throughout the internal granule cell layer, analyzed using a color deconvolution algorithm tuned to strong immunopositivity per the 3′3-diaminobenzidine chromogen, and averaged among the 20 boxes. pTDP-43 pathology in the frontal cortex and hippocampus was similarly quantified from high resolution images using ImageScope software. In the hippocampus and frontal cortex, traced regions of interest included the dentate fascia, the dentate fascia with the hippocampus proper (CA4-subiculum), and the cross-sectional midfrontal gyrus (spanning crest to sulcal depth). All images were subsequently processed with a color deconvolution algorithm specifically tuned to pTDP-43 immunoreactivity.

### Measurement of *C9ORF72* transcript variant 1 or transcript variant 3 mRNA

Total RNA was extracted from frozen cerebellum and frontal cortex using the RNeasy Plus mini kit (Qiagen), following manufacturer’s protocols. RNA quality and quantity were determined with an Agilent 2100 Bioanalyzer using the RNA Nano Chip (Agilent Technologies). High quality RNA was obtained from the cerebellum and frontal cortex of 49 and 48 patients, respectively. We performed a reverse transcription reaction with 300 ng of RNA as template, using an equal ratio of Random Hexamers and Oligo dT primers and the SuperScript III Kit (Invitrogen). Quantitative real-time PCR was then done on an ABI7900 PCR system (Applied Biosystems) using TaqMan gene expression assays (Life Technologies) in accordance with the manufacturer’s recommendations. We assessed neuronal markers synaptophysin (*SYP*; Hs00300531_m1) and microtubule-associated protein 2 (*MAP2*; Hs00258900_m1) as well as *C9ORF72* transcript variant 3 (NM_001256054.2; Hs00948764_m1). Using R Statistical Software (version 3.2.0; R Foundation for Statistical Computing), we calculated the median of replicates and took the geometric mean of neuronal markers, and subsequently determined expression levels relative to control subjects utilizing the ΔΔ Ct method. For transcript variant 1 (NM_145005.6), digital molecular barcoding (NanoString Technologies) was performed by the manufacturer using 100 ng of RNA as template. The NanoStringNorm R package and nSolver Analysis Software (NanoString Technologies, version 2.5) were used to account for technical assay variation using the geometric mean method. Maximum background subtraction was then performed, and data was normalized to the geometric mean of neuronal markers *SYP* and *MAP2*.

### Measurement of *C9ORF72* repeat size

For 53 patients for whom high quality DNA from the cerebellum and frontal cortex was available, the size of the G_4_C_2_ repeat expansion in these tissues was determined by Southern blotting as described elsewhere [36].

### Statistical analysis

In a cohort of 35 patients with the *C9ORF72* repeat expansion, associations of cerebellar poly(GP) levels quantified by immunoassay with poly(GP) immunopositivity quantified from immunostained cerebellar sections were evaluated using a Spearman’s test of correlation; Spearman’s correlation coefficient *r* and 95 % confidence intervals (CI) were estimated.

In our cohort of 55 patients, levels of poly(GP) were compared among neuroanatomical regions (i.e. cerebellum, frontal cortex, motor cortex, and hippocampus) using a Friedman rank sum test. This was followed by a paired Wilcoxon signed rank test for pairwise comparisons when significant differences were encountered (*p* < 0.0083 considered significant after Bonferroni correction). Associations between soluble and insoluble poly(GP) or poly(GA) levels in a given neuroanatomical region were evaluated using a Spearman’s test of correlation. The difference between soluble and insoluble poly(GP) as a percentage of total poly(GP) levels was evaluated using a Wilcoxon signed rank test, and the same was done for poly(GA).

For a given neuroanatomical area, we assessed associations of poly(GP) or poly(GA) with six variables [i.e. disease subgroups (i.e. ALS, FTLD, FTLD-MND), *C9ORF72* expansion size, *C9ORF72* variant 1, *C9ORF72* variant 3, age of onset, and survival after onset]. To adjust for multiple testing and control the family-wise error rate at 5 %, a Bonferroni correction was utilized separately for each outcome, and thus *p* < 0.0083 was considered significant. A Kruskal–Wallis rank sum test was performed to determine whether poly(GP) or poly(GA) levels differed significantly among disease subgroups, and when this test was significant, a Wilcoxon rank sum test was used for pairwise comparisons (*p* < 0.017 considered significant after Bonferroni correction). To assess associations between poly(GP) or poly(GA) levels and repeat size, *C9ORF72* transcript variant 1 or 3 expression, and age of onset of the *C9ORF72* expansion, a Spearman’s test of correlation was used. Associations between poly(GP) or poly(GA) levels and survival after disease onset were investigated using Cox proportional hazards regression models. Hazard ratios and 95 % CIs were estimated. Since all patients were deceased no censoring was necessary. Models were adjusted for age of onset and disease subgroup. Poly(GP) or poly(GA) levels were considered as dichotomous categorical variables using the median as the cut-off point.

In our cohort of 15 patients for whom cognitive status was evaluated, we assessed associations of seven outcomes [i.e. total poly(GP), soluble poly(GP), insoluble poly(GP), total poly(GA), soluble poly(GA), insoluble poly(GA), and pTDP-43 pathology] with two variables (i.e. cognitive score and neuropathological diagnosis). Associations of poly(GP), poly(GA) or pTDP-43 with cognitive score were assessed using Spearman’s test of correlation, and associations with neuropathological diagnosis were assessed using a Wilcoxon rank sum test (*p* < 0.025 considered significant after Bonferroni correction).

In a secondary analysis, associations between poly(GP) levels in all evaluated neuroanatomical regions and pTDP-43 pathology in the frontal cortex or hippocampus were analyzed using Spearman’s test of correlation (*p* < 0.025 considered significant after Bonferroni correction). Associations between cerebellar poly(GA) levels and pTDP-43 pathology in the frontal cortex or hippocampus were similarly assessed. A Wilcoxon rank sum test was used for pairwise comparisons between ALS and FTLD-MND subgroups in the clinical cohort with respect to sex, age of onset, survival after onset, education, and cognitive score (*p* < 0.01 considered significant after Bonferroni correction).

## Results

### Soluble and insoluble poly(GP) are detected in various neuroanatomical regions and are highest in the cerebellum

**Fig. 1:**
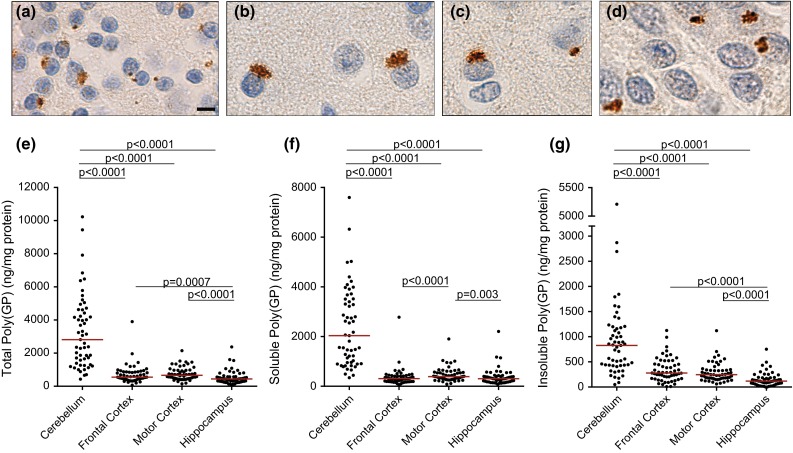
Comparison of poly(GP) levels in different neuroanatomical regions of *C9ORF72* mutation carriers. **a**–**d** Poly(GP)-immunoreactive inclusions in the cerebellum (**a**), frontal cortex (**b**), motor cortex (**c**) and hippocampus (**d**) of a *C9ORF72* repeat expansion carrier. *Scale bar* 5 µm. **e**–**g** Levels of total poly(GP) (**e**), soluble poly(GP) (**f**), or insoluble poly(GP) (**g**) in cerebellum, frontal cortex, motor cortex, and hippocampus of patients with *C9ORF72* repeat expansions (*n* = 55). The median in a given group is denoted by a *solid horizontal line*

We previously reported, and show in Fig. 1a–d, that poly(GP) inclusions are detected in abundance in the cerebellum, neocortex, and hippocampus of *C9ORF72* mutation carriers by immunohistochemistry [3]. In the present study, we utilized an immunoassay to measure levels of poly(GP) in various neuroanatomical regions. A comparison of cerebellar poly(GP) levels determined using this biochemical approach with poly(GP) immunopositivity in stained cerebellar sections from 35 *C9ORF72* mutation carriers revealed a significant correlation between these two measures (Supplementary Table 2). One of the advantages of biochemical analyses of poly(GP) burden is the ability to measure different forms of the protein in a quantitative manner. Since soluble protein oligomers, like insoluble forms of aggregating proteins, may be pathogenic, we measured both soluble and insoluble poly(GP) levels in cerebellum, frontal cortex, motor cortex, and hippocampus homogenates sequentially extracted with RIPA buffer (soluble fraction) and urea buffer (insoluble fraction). As shown in Fig. 1e–g, levels of soluble and insoluble poly(GP) were significantly higher in the cerebellum compared to the other neuroanatomical regions. Given that a significant correlation existed between these forms of poly(GP) in all regions (Supplementary Table 3), the analyses presented below are limited to total poly(GP) levels for simplicity. Data on soluble and insoluble poly(GP) can be found in the Electronic Supplementary Material (Supplementary Tables 4–8).

### Cerebellar poly(GP) levels are significantly lower in *C9ORF72* mutation carriers with ALS compared to patients with FTLD or FTLD-MND

**Fig. 2:**
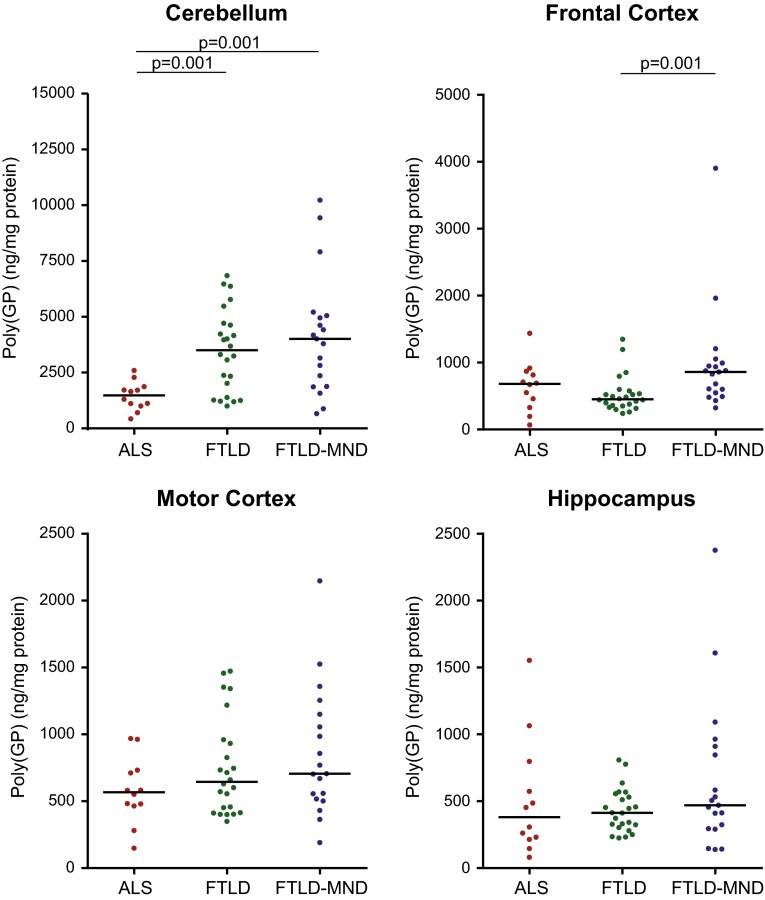
Poly(GP) levels are significantly lower in the cerebellum of *C9ORF72* mutation carriers with ALS compared to patients with FTLD or FTLD-MND. Shown are total levels of poly(GP) in the indicated neuroanatomical regions by disease subgroup [amyotrophic lateral sclerosis (ALS), *n* = 12; frontotemporal lobar degeneration (FTLD), *n* = 24; and FTLD with motor neuron disease (FTLD-MND); *n* = 19]. Poly(GP) levels in the cerebellum were significantly lower in patients with ALS compared to patients with FTLD or FTLD-MND. In the frontal cortex, poly(GP) levels were significantly higher in the FTLD-MND subgroup compared to the FTLD subgroup. The median in a given group is denoted by a *solid horizontal line*

We next evaluated whether poly(GP) levels in the cerebellum, frontal cortex, motor cortex, or hippocampus differ among patients neuropathologically diagnosed with ALS (*n* = 12), FTLD (*n* = 24), or FTLD-MND (*n* = 19). Of interest, poly(GP) levels in the cerebellum were significantly lower in the ALS subgroup (median 1481 ng/mg, IQR 1083–1758) compared to the FTLD (median 3501 ng/mg, IQR 1864–4652, *p* = 0.001) or FTLD-MND (median 4014 ng/mg, IQR 2119–5002, *p* = 0.001) subgroups (Table 2; Fig. 2). In the frontal cortex, poly(GP) levels also differed based on neuropathological subtype, although the expression profile varied from that observed in the cerebellum; patients with FTLD-MND (median 859 ng/mg, IQR 575–971) had significantly higher poly(GP) levels in the frontal cortex compared to patients with FTLD (median 452 ng/mg, IQR 358–546, *p* = 0.001) (Table 2; Fig. 2). No differences in poly(GP) levels among disease subgroups were detected in the motor cortex or hippocampus (Table 2; Fig. 2).

**Table 2:** Comparison of poly(GP) levels among disease subgroups

	*p* value*	ALS vs. FTLD			ALS vs. FTLD-MND			FTLD vs. FTLD-MND		
		ALS	FTLD	*p* value	ALS	FTLD-MND	*p* value	FTLD	FTLD-MND	*p* value
Cerebellum	**0.001**	1481 (1083–1758)	3501 (1864–4652)	**0.001**	1481 (1083–1758)	4014 (2119–5002)	**0.001**	3501 (1864–4652)	4014 (2119–5002)	0.57
Frontal cortex	**0.004**	682 (428–829)	452 (358–546)	0.19	682 (428–829)	859 (575–971)	0.15	452 (358–546)	859 (575–971)	**0.001**
Motor cortex	0.27	567 (476–717)	645 (443–940)	–	567 (476–717)	706 (537–1104)	–	645 (443–940)	706 (537–1104)	–
Hippocampus	0.52	381 (227–631)	414 (319–538)	–	381 (227–631)	470 (310–879)	–	414 (319–538)	470 (310–879)	–

Poly(GP) levels presented as median (IQR)

In total, associations with six variables were examined (i.e. disease subgroups, *C9ORF72* expansion size, *C9ORF72* variant 1, *C9ORF72* variant 3, age of onset, and survival after onset), and therefore *p* values lower than 0.0083 were considered significant after Bonferroni correction

Of note, only one of those variables is shown in this table (i.e. disease subgroups)

*p* values denoted by bold remain significant after adjustment for multiple testing

*ALS* amyotrophic lateral sclerosis, *FTLD* frontotemporal lobar degeneration, *FTLD-MND* frontotemporal lobar degeneration with motor neuron disease

* A Kruskal–Wallis rank sum test was performed to determine whether poly(GP) levels differed among disease subgroups (*p* < 0.0083 considered significant after Bonferroni correction); when significant differences were detected a Wilcoxon rank sum test was used for pairwise comparisons (*p* < 0.017 considered significant after Bonferroni correction)

### Cerebellar poly(GP) associates with *C9ORF72* transcript variant 3 expression but not with expression of variant 1 or with repeat size

**Fig. 3:**
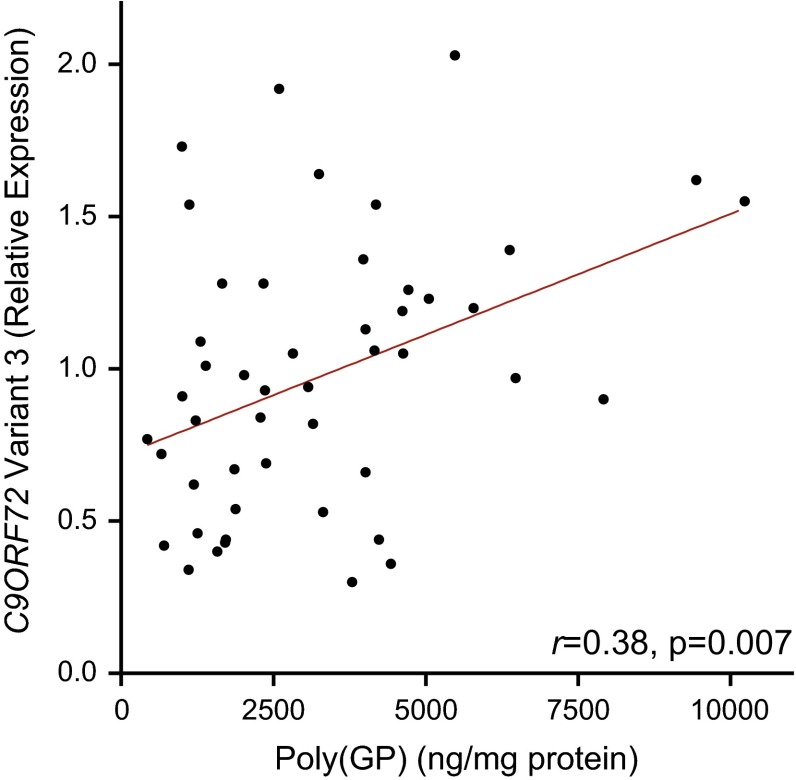
Cerebellar poly(GP) associates with *C9ORF72* transcript variant 3 expression. Associations of total poly(GP) levels in the cerebellum with expression of *C9ORF72* transcript variant 3 (*n* = 49). The *straight line* is a linear regression line

Because our studies uncovered associations between cerebellar or frontal cortical poly(GP) and the neuropathological diagnosis of *C9ORF72* patients, we sought to identify factors that influence poly(GP) levels. We first investigated associations between poly(GP) and repeat size of the *C9ORF72* expansion and found no association between these two variables in either the cerebellum or the frontal cortex (Table 3). We next evaluated associations between poly(GP) and transcript levels for *C9ORF72* variant 1 or variant 3. The G_4_C_2_ repeat is located between exons 1a and 1b; if exon 1a is used, as is the case for variants 1 and 3, the repeat is located in intron 1 and is transcribed. If exon 1b is used, the repeat is located in the promoter region and is not transcribed (variant 2; NM_018325.4). In the cerebellum, poly(GP) levels associated with variant 3 (*r* 0.38, 95 % CI 0.10–0.62, *p* = 0.007; Fig. 3) but not with variant 1 (Table 3). In the frontal cortex, no association between poly(GP) levels and *C9ORF72* transcript variant 1 or variant 3 was detected (Table 3).

**Table 3:** Associations of total poly(GP) levels with *C9ORF72* expansion size, *C9ORF72* variants 1 and 3, and age at onset

	Spearman’s *r* (95 % CI)	*p* value
Cerebellum		
*C9ORF72* expansion size	−0.19 (−0.47 to 0.11)	0.17
*C9ORF72* variant 1	0.21 (−0.08 to 0.48)	0.14
*C9ORF72* variant 3	0.38 (0.10 to 0.62)	**0.007**
Age at onset	0.02 (−0.28 to 0.30)	0.91
Frontal cortex		
*C9ORF72* expansion size	−0.09 (−0.35 to 0.18)	0.50
*C9ORF72* variant 1	0.13 (−0.17 to 0.41)	0.37
*C9ORF72* variant 3	0.27 (−0.03 to 0.52)	0.07
Age at onset	−0.28 (−0.56 to 0.005)	0.04

Spearman’s *r* correlation coefficients, 95 % confidence intervals (CIs), and *p* values are presented. In total, associations with six variables were examined (i.e. disease subgroups, *C9ORF72* expansion size, *C9ORF72* variant 1, *C9ORF72* variant 3, age of onset, and survival after onset), and therefore *p* values lower than 0.0083 were considered significant after Bonferroni correction

Of note, only four of those variables are shown in this table

*p* values denoted by bold remain significant after adjustment for multiple testing

### Cerebellar poly(GP) associates with cognitive impairment

Despite the associations between poly(GP) in the cerebellum or frontal cortex and neuropathological subgroup, poly(GP) levels in these neuroanatomical regions did not associate with age of disease onset in our overall cohort of patients (Table 3). Furthermore, survival after onset, adjusted for age of onset and disease subgroup, was not associated with poly(GP) in these regions (Supplementary Table 6).

Since poly(GP) levels could nevertheless influence the clinical phenotype of patients, we examined associations between poly(GP) levels in different neuroanatomical regions and cognitive impairment. These studies were carried out on 15 c9ALS patients for whom neuropsychological data was available (Table 4; Supplementary Table 1). Ten of the 15 patients in this clinical cohort presented with ALS (67 %), four with ALS accompanied by cognitive impairment (27 %), and one with behavioral variant FTD (bvFTD-ALS, 7 %; Supplementary Table 1). At autopsy, nine patients were neuropathologically diagnosed with ALS (60 %) and six with FTLD-MND (40 %). No significant differences in sex, age of disease onset, survival after onset, education, or cognitive score were observed between the ALS and FTLD-MND subgroups (Table 4).

**Table 4:** Characteristics of patients in the clinical cohort

	Overall cohort (*n* = 15)	ALS (*n* = 9)	FTLD-MND (*n* = 6)	*p* value ALS vs. FTLD-MND
Women	8 (53 %)	6 (66 %)	2 (33 %)	0.31
Age of onset (years)	58 (51–66)	52 (49–67)	61 (58–65)	0.55
Survival after onset (years)	2.3 (1.7–3.7)	1.8 (1.5–3.7)	2.5 (2.2–3.4)	0.60
Education (years)	15 (14–17*)	14 (13–16)	16 (14–19)	0.35
Cognitive score	1.25 (0.25–2.00)	0.50 (0.00–1.50)	2.00 (1.44–2.00)	0.02

Data presented as median (IQR) or number (%)

Percentages may not sum to 100 % because of rounding

*p* values lower than 0.01 were considered significant after Bonferroni correction

*ALS* amyotrophic lateral sclerosis, *FTLD-MND* frontotemporal lobar degeneration with motor neuron disease

* Education was assumed to be 19 years for three patients with a degree of MD, DDS, or JD

**Fig. 4:**
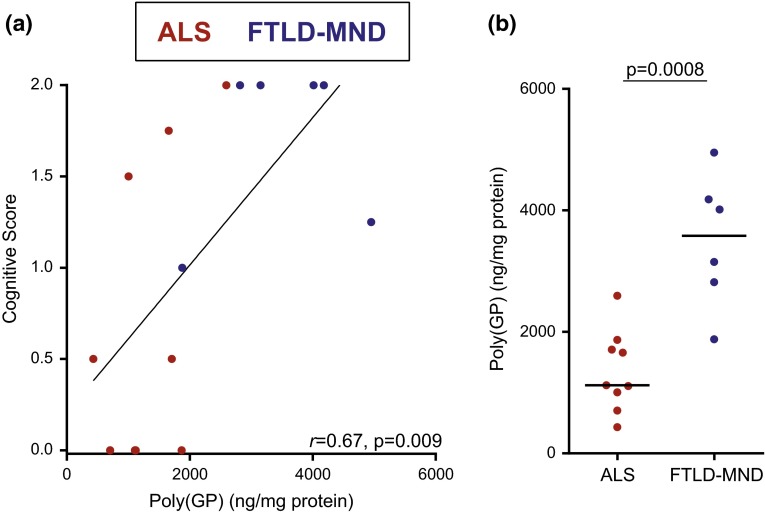
Cerebellar poly(GP) associates with cognitive score and differs based on neuropathological subgroup in the clinical cohort of *C9ORF72* mutation carriers. Four evaluators assessed cognitive function of patients in the clinical cohort (*n* = 15) from independent review of clinical records and test results, and assigned to each patient a score (0 = normal, 1 = mild or questionable impairment, or 2 = definitely abnormal). The final cognitive score for each patient is the average of the scores assigned by the four evaluators. Associations of total poly(GP) in the cerebellum with cognitive score (**a**) or neuropathological diagnosis (**b**) of patients are shown. In panel **a**, the *straight line* is a linear regression line. In panel **b**, the median in a given group is denoted by a *solid horizontal line*. *Red circles* represent patients neuropathologically diagnosed with amyotrophic lateral sclerosis (ALS), and *blue circles* represent patients with frontotemporal lobar degeneration with motor neuron disease (FTLD-MND)

Notably, an association between poly(GP) levels in the cerebellum and cognitive score was detected (*r* = 0.67, 95 % CI 0.22–0.88, *p* = 0.009) (Table 5; Fig. 4a). In contrast, poly(GP) levels in frontal cortex, motor cortex, and hippocampus did not associate with cognitive score (Table 5). As anticipated, an association between pTDP-43 pathology in the frontal cortex and cognitive score was observed (*r* = 0.58, 95 % CI 0.09–0.85, *p* = 0.025) (Table 5).

**Table 5:** Associations of total poly(GP) levels or pTDP-43 pathology with cognitive score or neuropathological diagnosis in the clinical cohort

	Cognitive score		Neuropathological diagnosis (ALS vs. FTLD-MND)		
	Spearman’s *r* (95 % CI)	*p* value	ALS	FTLD-MND	*p* value
Poly(GP) levels					
Cerebellum	0.67 (0.22 to 0.88)	**0.009**	1122 (1005–1708)	3583 (2901–4139)	**0.0008**
Frontal cortex	−0.01 (−0.53 to 0.52)	0.90	692 (460–815)	771 (621–918)	0.33
Motor cortex	0.18 (−0.38 to 0.64)	0.51	553 (464–582)	781 (678–954)	0.05
Hippocampus	0.09 (−0.45 to 0.59)	0.74	308 (231–486)	558 (513–780)	0.07
pTDP-43 pathology					
Frontal cortex	0.58 (0.09 to 0.85)	**0.03**	0.0008 (0.0004–0.001)	0.003 (0.002–0.008)	**0.01**
Hippocampus	0.50 (−0.03 to 0.81)	0.06	0.0005 (0.0002–0.001)	0.006 (0.003–0.01)	**0.01**

Spearman’s *r* correlation coefficients, 95 % confidence intervals (CIs), median (IQR), and *p* values are presented

Units for median values of poly(GP) levels are ng/mg protein

pTDP-43 pathology was quantified from sections immunostained with anti-pTDP-43 followed by measurement of immunopositivity of scanned images using a color deconvolution algorithm; median values for pTDP-43 are in arbitrary units

Given the assessment of poly(GP) levels or pTDP-43 pathology with two variables (i.e. cognitive score and neuropathological diagnosis), *p* values lower than 0.025 were considered significant after Bonferroni correction

*p* values denoted by bold remain significant after adjustment for multiple testing

*ALS* amyotrophic lateral sclerosis, *FTLD-MND* frontotemporal lobar degeneration with motor neuron disease

Similarly to what was observed in the 55 *C9ORF72* mutation carriers, cerebellar poly(GP) levels in patients with ALS (median 1122 ng/mg, IQR 1005–1708) were significantly lower compared to patients with FTLD-MND (median 3583 ng/mg, IQR 2901–4139, *p* = 0.0008) in the smaller clinical cohort (Table 5; Fig. 4b). In the other neuroanatomical regions, no difference in poly(GP) levels was detected between neuropathological subgroups (Table 5). As would be expected, pTDP-43 pathology in frontal cortex and hippocampus was significantly more abundant in the FTLD-MND subgroup compared to the ALS subgroup (Table 5).

We additionally investigated potential relationships between c9RAN protein expression and pTDP-43 pathology. No association between poly(GP) levels in the frontal cortex or hippocampus and pTDP-43 pathology in these same regions was detected. Likewise, poly(GP) levels in the motor cortex did not associate with frontal cortical or hippocampal pTDP-43 pathology. However, in line with our findings that poly(GP) levels in the cerebellum associated with neuropathological diagnosis, as did pTDP-43 pathology in the frontal cortex and hippocampus, we detected an association of cerebellar poly(GP) levels with pTDP-43 pathology in the frontal cortex (*r* = 0.69, 95 % CI 0.22–0.92, *p* = 0.005) or hippocampus (*r* = 0.69 95 % CI 0.25–0.89, *p* = 0.005) (Supplementary Table 8).

### Associations of cerebellar poly(GA) with neuropathological and clinical features

**Fig. 5:**
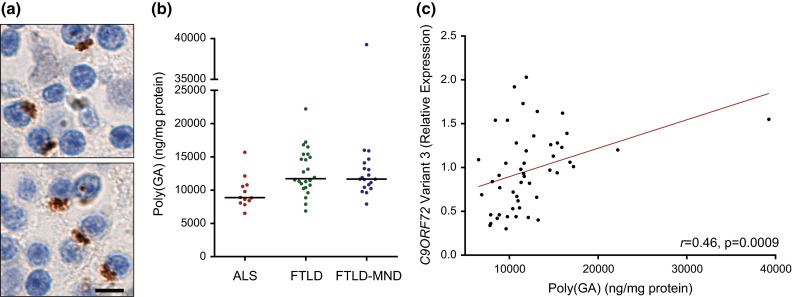
Cerebellar poly(GA) levels in *C9ORF72* mutation carriers with ALS, FTLD or FTLD-MND, and associations between poly(GA) and *C9ORF72* transcript variant 3 expression. **a** Poly(GA)-immunoreactive inclusions in the cerebellum of a *C9ORF72* repeat expansion carrier. *Scale bar* 5 µm. **b** Shown are total levels of cerebellar poly(GA) by disease subgroup [amyotrophic lateral sclerosis (ALS), *n* = 12; frontotemporal lobar degeneration (FTLD), *n* = 24; and FTLD with motor neuron disease (FTLD-MND), *n* = 19]. The median in a given group is denoted by a *solid horizontal line*. **c** Associations of poly(GA) levels in the cerebellum with expression of *C9ORF72* transcript variant 3 (*n* = 49). The *straight line* is a linear regression line

**Fig. 6:**
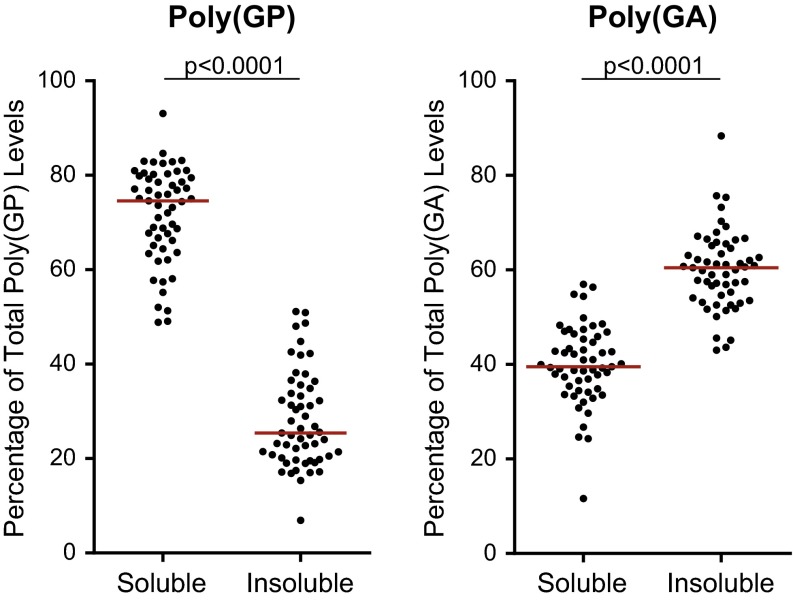
Solubility profiles of poly(GP) and poly(GA) in the cerebellum. Comparison of soluble and insoluble poly(GP) or poly(GA) in the cerebellum of patients with *C9ORF72* repeat expansions (*n* = 55). Note that soluble poly(GP) levels were significantly higher than insoluble poly(GP) levels, whereas soluble poly(GA) levels were significantly lower than insoluble poly(GA) levels. The median in a given group is denoted by *a solid horizontal line*

Given our findings above and the fact that poly(GA) pathology is abundant in the cerebellum (Fig. 5a), and given a recent report showing that poly(GA) inclusion burden in cerebellar granule cells correlated with neuropathological subgroup [30], we investigated whether cerebellar poly(GA) levels assessed by immunoassay differ based on neuropathological diagnosis or cognitive score in our case series. As observed for cerebellar poly(GP), there existed a significant correlation between soluble and insoluble poly(GA) levels in the cohort of 55 *C9ORF72* mutation carriers (Supplementary Table 9). However, the solubility profile of poly(GA) differed from that of poly(GP); while soluble poly(GP) levels were significantly higher than insoluble poly(GP) levels in the cerebellum, the opposite was observed for poly(GA) (Fig. 6).

Upon comparing cerebellar poly(GA) among neuropathological subgroups, differences in poly(GA) levels among subgroups neared statistical significance but the p value (*p* = 0.0088) did not meet the cut-off (*p* < 0.0083) established by Bonferroni correction. Nevertheless, total poly(GA) levels trended lower in the ALS subgroup (median 8877 ng/mg, 8327–10,616) in comparison to the FTLD (median 11,723 ng/mg, 10,666–15,054) or FTLD-MND (median 11,677 ng/mg, 10,627–13,673) subgroups (Fig. 5b; Supplementary Table 10). Compared to cerebellar poly(GP), the difference in poly(GA) levels among subgroups was less robust. Whereas poly(GP) levels in FTLD and FTLD-MND were approximately 2.5-fold higher than in ALS (Fig. 2), a 1.3-fold increase in poly(GA) levels was detected in FTLD and FTLD-MND compared to ALS (Fig. 5b).

Consistent with total poly(GP) in the cerebellum, total poly(GA) levels associated with *C9ORF72* variant 3 (*r* 0.46, 95 % CI 0.22–0.66, *p* = 0.0009; Fig. 5c) but not with *C9ORF72* variant 1, repeat size, age of onset, or survival after onset (Supplementary Tables 11 and 12). In contrast to cerebellar poly(GP), total poly(GA) levels did not associate with cognitive score or with neuropathological diagnosis (ALS vs. FTLD-MND) in our clinical cohort of 15 patients (Supplementary Table 13). An association between soluble poly(GA) and neuropathological diagnosis was nonetheless detected in this smaller cohort (Supplementary Table 13). Furthermore, poly(GA) levels in the cerebellum associated with pTDP-43 pathology in the frontal cortex or hippocampus (Supplementary Table 14), as did cerebellar poly(GP).

## Discussion

Neuropathologic and clinical diversity characterize *C9ORF72* repeat expansion carriers, but the molecular basis for this variation remains elusive. Our studies reveal intriguing findings involving c9RAN proteins in the cerebellum. We show that cerebellar poly(GP) levels were significantly lower in ALS patients compared to FTLD and FTLD-MND patients. This is not likely due to the shorter disease duration of ALS, which could afford less time for c9RAN proteins to accumulate, since poly(GP) levels in the frontal cortex, motor cortex, and hippocampus did not similarly differ between ALS and FTLD or FTLD-MND subgroups. Rather, the results suggest that expression of the *C9ORF72* repeat expansion in cerebellum influences neuropathological phenotypes and this may consequently influence clinical features. Indeed, we found that cerebellar poly(GP) levels associated with cognitive impairment. Whether this occurs as a direct result of poly(GP) accumulation, or whether poly(GP) serves as a marker for aberrant events triggered by *C9ORF72* repeat expansions, is not yet known. Similarly to our observations with poly(GP), cerebellar poly(GA) levels trended lower in patients with ALS compared to patients with FTLD or FTLD-MND, but the p value narrowly exceeded the threshold of significance after adjustment for multiple comparisons (*p* < 0.0083). This is likely due to the fact that differences in poly(GA) levels among disease subgroups were less robust than those observed for poly(GP), an observation that may also explain the lack of association between poly(GA) and cognitive impairment in our clinical cohort. A larger number of patients may be required to detect potential associations between poly(GA) levels and neuropathological diagnosis or cognitive score. Indeed, while findings in our retrospectively identified clinical cohort are of particular interest, further validation in larger cohorts of c9ALS and c9FTD patients in whom detailed cognitive and behavioral data are available is warranted in order to define contributions of c9RAN proteins to clinical phenotypes.

Our study supports the need for more sensitive and quantitative approaches when evaluating the relationship between putatively pathologic proteins and disease phenotypes. Previous studies that utilized semi-quantitative grading systems of c9RAN protein pathology, in particular for poly(GA), did not detect associations with clinical or neuropathological features [9, 16, 18]. It must be recognized, however, that semi-quantitative analyses may not be sufficiently sensitive to discern differences among patient subgroups, especially when cohorts are small. More recently, using a quantitative analysis of inclusion pathology, Schuldi et al. reported that inclusions of poly(GA) are less frequent in cerebellar granule cells of patients with ALS (*n* = 3) and FTLD-MND (*n* = 8) compared to patients with FTLD (*n* = 3) [30]. This data differs from our observations that cerebellar poly(GA) trended lower in patients with ALS (*n* = 12) compared to patients with FTLD (*n* = 24) as well as patients with FTLD-MND (*n* = 19). Yet, it must be kept in mind that the two studies used different case series and different methods to quantify poly(GA) burden.

The impetus for employing a biochemical approach to evaluate c9RAN proteins in this study was that it allows for non-biased, sensitive, and quantitative measures, and may permit the detection of a distinct pool of protein species not discernable by immunohistochemistry. With respect to the latter, we examined soluble and insoluble poly(GP) and poly(GA), finding a strong correlation between the soluble and insoluble forms of each of these c9RAN proteins. We additionally observed that poly(GP) and poly(GA) have different solubility profiles in brain tissue. A greater percentage of total poly(GP) was soluble, while a greater percentage of total poly(GA) was insoluble. Whether and how this influences the toxic potential of these c9RAN proteins is not yet known but worthy of investigation. Our data also suggest that poly(GA) levels are higher than levels of poly(GP); however, a direct comparison in the abundance of these two proteins should be made with some caution given the different biochemical properties of the proteins, differences in affinities of the poly(GP) and poly(GA) antibodies used for the immunoassays, and differences in the calibrators used for interpolating protein concentrations.

Little is known about factors that govern c9RAN protein expression. We detected an association between cerebellar poly(GP) or poly(GA) levels and *C9ORF72* transcript variant 3, the pre-mRNA of which contains the expanded repeat that serves as a template for RAN translation. Surprisingly, the same association was not observed for transcript variant 1. Although pre-mRNA of variant 1 also contains the repeat expansion, one could speculate that the absence of an association may be a reflection of unique properties of each transcript variant (i.e. predisposition to RAN translation), which requires further study. There was no association between poly(GP) or poly(GA) levels in the cerebellum or frontal cortex with repeat size. This is congruent with our previous report that repeat size does not differ between disease subgroups [36], in contrast to what we show here for cerebellar poly(GP) levels. It is also worth noting that poly(GP) expression is higher in the cerebellum compared to the frontal cortex (Fig. 1), whereas repeat lengths in the cerebellum are smaller than those in the frontal cortex [36].

Of importance, the findings herein add to a growing body of evidence supporting cerebellar involvement in c9FTD/ALS. For instance, we discovered far more transcriptome changes in the cerebellum than frontal cortex of c9ALS patients [27], and cerebellar atrophy is reported in *C9ORF72* expansion carriers with neuroimaging methods [17, 32, 38]. Furthermore, with regards to the association of cerebellar poly(GP) with cognitive impairment, neuroanatomical studies have revealed reciprocal cerebellar connectivity with areas of the cerebral cortex involved in higher cognitive functioning. Output projections from the cerebellum first synapse on the dentate nucleus and then the thalamus before projecting to the cerebral cortex. Conversely, the cerebral cortex connects to the cerebellum via projections that synapse on the pons [7]. It is also notable that cerebellar activation during cognitive tasks has been recorded by functional neuroimaging [24], and that neuropsychological studies on patients with cerebellar lesions have identified cognitive and affective disturbances, leading to the concept of “cerebellar cognitive affective syndrome” [31]. A large spectrum of deficits has been described in patients with cerebellar damage, including executive dysfunction, mild language symptoms, blunting of affect, disinhibition, obsessive–compulsive traits, and psychosis. Patients with *C9ORF72* repeat expansions present with many similar features [29].

Whereas TDP-43 pathology is reportedly consistent in FTLD and ALS patients with and without the *C9ORF72* repeat expansion [9], neuropsychiatric symptoms, as well as cognitive and behavioral impairment, may be more common in FTD and ALS patients that carry the *C9ORF72* mutation compared to non-carriers [8, 29]. Features unique to the *C9ORF72* repeat expansion, such as c9RAN proteins, may thus contribute to the increased frequency of certain phenotypes in c9FTD/ALS. Indeed, we noted that one ALS patient with definite cognitive impairment, who was neuropathologically diagnosed with ALS, had sparse extramotor TDP-43 pathology but relatively high cerebellar poly(GP) levels (Fig. 4). In a similar manner, abundant poly(GA) pathology in cerebral cortical regions, hippocampus, and cerebellum, but only sparse TDP-43 pathology, have been reported in three *C9ORF72* repeat expansion patients who developed fairly rapid cognitive decline, but died prematurely due to unrelated illnesses [4]. While data from our group and others [4, 26], indicate that clinical features can develop in *C9ORF72* repeat expansion carriers with c9RAN protein pathology but limited TDP-43 pathology, we did nonetheless uncover a correlation between c9RAN proteins and pTDP-43. While levels of poly(GP) and poly(GA) in the frontal cortex or hippocampus did not associate with pTDP-43 pathology in these same regions, there were significant associations of poly(GP) or poly(GA) levels in the cerebellum with pTDP-43 pathology in the frontal cortex or hippocampus. These findings could point toward a relationship whereby cerebellar c9RAN proteins influence TDP-43 in the frontal cortex and hippocampus, or simply reflect the fact that cerebellar c9RAN protein levels and pTDP-43 pathology in the frontal cortex and hippocampus independently associate with neuropathological diagnosis and consequently associate with each other. Nevertheless, the finding that poly(GA) levels in cerebellum associated with pTDP-43 pathology in the frontal cortex adds strength to the notion that, like poly(GP), cerebellar poly(GA) differs based on neuropathological subgroup.

Overall, our findings implicate cerebellar abnormalities as a contributor to the neuropathological and clinical heterogeneity associated with the *C9ORF72* repeat expansion, and support additional investigations on the role of this largely overlooked neuroanatomical region in c9FTD/ALS.

## Electronic supplementary material

Supplementary material 1 (DOCX 82 kb)
