# Supplementary material for: Cerebellar c9RAN proteins associate with clinical and neuropathological characteristics of C9ORF72 repeat expansion carriers
Source: Acta Neuropathol. 2015 Sep 8;130(4):559–73. doi: 10.1007/s00401-015-1474-4 (PMC4575385; doi:10.1007/s00401-015-1474-4)
Supplement: Supplementary file 1 — Supplementary material 1 (DOCX 82 kb) [file 401_2015_1474_MOESM1_ESM.docx]

**Electronic Supplementary Material**

**Cerebellar c9RAN proteins associate with clinical and neuropathological characteristics of *C9ORF72* repeat expansion carriers**

*Tania F. Gendron^1*^, Marka van Blitterswijk^1*^, Kevin F. Bieniek^1,2^, Lillian M. Daughrity^1^, Jie Jiang^3^, Beth K. Rush^4^, Otto Pedraza^4^, John A. Lucas^4^, Melissa E. Murray^1^, Pamela Desaro^5^, Amelia Robertson^5^, Karen Overstreet^5^, Colleen S. Thomas^6^, Julia E. Crook^6^, Monica Castanedes-Casey^1^, Linda Rousseau^1^*, *Keith A. Josephs^7^, Joseph E. Parisi^7^, David S. Knopman^7^, Ronald C. Petersen^7^, Bradley F. Boeve^7^, Neill R. Graff-Radford^5^, Rosa Rademakers^1^, Clotilde Lagier-Tourenne^3,8^, Dieter Edbauer^9,10,11^, Don W. Cleveland^3,12^, Dennis W. Dickson^1^, Leonard Petrucelli^1^, and Kevin B. Boylan^5^*

**Affiliations:**

*^1^*Department of Neuroscience, Mayo Clinic, Jacksonville, FL 32224, USA

*^2^*Mayo Graduate School, Mayo Clinic, Rochester, MN 55905, USA

*^3^*Ludwig Institute, University of California at San Diego, La Jolla, CA 92093, USA

*^4^*Department of Psychiatry and Psychology, Mayo Clinic, Jacksonville, FL 32224, USA

*^5^*Department of Neurology, Mayo Clinic, Jacksonville, FL 32224, USA

*^6^*Section of Biostatistics, Mayo Clinic, Jacksonville, FL 32224, USA

*^7^*Department of Neurology, Mayo Clinic, Rochester, MN 55905, USA

*^8^*Department of Neurosciences, University of California at San Diego, La Jolla, CA 92093, USA

*^9^*German Center for Neurodegenerative Diseases (DZNE), Munich, Germany

*^10^*Institute for Metabolic Biochemistry, Ludwig-Maximilians University Munich, Munich, Germany

*^11^*Munich Cluster of Systems Neurology (SyNergy), Munich, Germany

*^12^*Department of Cellular and Molecular Medicine, University of California at San Diego, La Jolla, CA 92093, USA

* Tania F. Gendron, PhD, and Marka van Blitterswijk, PhD, contributed equally to this work.

**Correspondence to:**

Kevin B. Boylan, MD

Department of Neurology, Mayo Clinic Florida

Telephone number: +1 904-953-8215

Fax number: +1 904-953-6036

Email: [Boylan.Kevin@mayo.edu](mailto:Boylan.Kevin@mayo.edu)

Leonard Petrucelli, PhD

Department of Neuroscience, Mayo Clinic Florida,

Telephone number: +1 904-953-2855

Fax number: +1 904-953-6276

Email: [Petrucelli.Leonard@mayo.edu](mailto:Petrucelli.Leonard@mayo.edu)

**Supplementary Tables**

| **Supplementary Table 1: Characteristics of patients in the clinical cohort** | | | | | | | | | |
| --- | --- | --- | --- | --- | --- | --- | --- | --- | --- |
| **ID** | **Gender** | **Education**  **(years)** | **Family**  **history^1^** | **Age**  **of onset** | **ALS**  **onset**  **site** | **Survival**  **(years)** | **Clinical**  **diagnosis^2^** | **Autopsy**  **diagnosis** | **Cognitive**  **score** |
| 1 | M | 14 | ? FTD^3^ | 57 | B | 2.3 | bvFTD-ALS | FTLD-MND | 2.00^B^ |
| 2 | M | 16 | ALS | 52 | UE | 6.3 | ALS | ALS | 0.00 |
| 3 | M | Post grad | ALS | 57 | UE | 3.7 | ALS | FTLD-MND | 2.00^S^ |
| 4 | F | Post grad | None | 49 | LE | 1.0 | ALS | ALS | 0.00^S^ |
| 5 | M | Post grad | None | 66 | UE | 1.7 | ALS | FTLD-MND | 1.00^S^ |
| 6 | F | 14 | -- | 41 | LE | 1.2 | ALS | ALS | 0.00 |
| 7 | F | 12 | ALS | 45 | LE | 3.7 | ALS-FTCI | ALS | 1.50 |
| 8 | F | 15 | ? FTD^3^ | 65 | UE | 2.6 | ALS | FTLD-MND | 2.00 ^S^ |
| 9 | M | 13 | ? FTD^3^ | 49 | B | 4.1 | ALS-FTCI | ALS | 2.00 ^S,B^ |
| 10 | F | 14 | FTLD^4^ | 66 | B | 1.7 | ALS | ALS | 0.50^S^ |
| 11 | F | 18 | ALS;  ? FTD^3^ | 63 | B | 3.7 | ALS | FTLD-MND | 2.00^S^ |
| 12 | M | 16 | None | 70 | LE | 1.6 | ALS-FTCI | ALS | 1.75 |
| 13 | F | 8 | ALS;  ? FTD^3^ | 64 | B | 1.4 | ALS | ALS | 0.50^S^ |
| 14 | M | 12 | ALS | 58 | UE | 2.1 | ALS-FTCI | FTLD-MND | 1.25 |
| 15 | F | 16 | None | 68 | LE | 3.1 | ALS | ALS | 0.00 |
| ^1^Diagnoses in first- or second-degree relative(s).  ^2^Clinical diagnosis at presentation. ^3^Dementia suggestive of FTD lacking confirmation. ^4^Autopsy confirmed diagnosis. ^S^ALS Cognitive Behavioral Screen (ALS-CBS) data available. ^B^Neuropsychological test battery data available. ALS=amyotrophic lateral sclerosis. B=bulbar. bvFTD=behavioral variant FTD. FTCI=frontotemporal cognitive impairment. FTD=frontotemporal dementia. FTLD-MND=frontotemporal lobar degeneration with motor neuron disease. LE=lower extremity. UE=upper extremity. | | | | | | | | | |

| Supplementary Table 2: Associations of poly(GP) levels with poly(GP) immunopositivity in a cohort of 35 *C9ORF72* repeat expansion carriers | | |
| --- | --- | --- |
|  | **Poly(GP) immunohistochemistry** | |
| Poly(GP) immunoassay | **Spearman’s r (95% CI)** | **p value** |
| Soluble poly(GP) | 0.49 (0.15 to 0.75) | **0.003** |
| Insoluble poly(GP) | 0.47 (0.09 to 0.76) | **0.004** |
| Total poly(GP) | 0.48 (0.10 to 0.76) | **0.004** |
| Spearman’s r correlation coefficients, 95% confidence intervals (CIs), and p values are presented. | | |

| Supplementary Table 3: Associations between soluble and insoluble poly(GP) levels in different neuroanatomical regions | | |
| --- | --- | --- |
|  | **Spearman’s r (95% CI)** | **p value** |
| Soluble vs. insoluble poly(GP) in the full cohort (n=55) |  |  |
| Cerebellum | 0.82 (0.70 to 0.89) | **2.03e-14** |
| Frontal Cortex | 0.57 (0.34 to 0.74) | **5.10e-06** |
| Motor Cortex | 0.61 (0.40 to 0.77) | **7.41e-07** |
| Hippocampus | 0.63 (0.42 to 0.80) | **2.08e-07** |
| Soluble vs. insoluble poly(GP) in the clinical cohort (n=15) |  |  |
| Cerebellum | 0.89 (0.65 to 0.97) | **9.09e-06** |
| Frontal Cortex | 0.55 (0.02 to 0.88) | **0.03** |
| Motor Cortex | 0.74 (0.31 to 0.95) | **0.002** |
| Hippocampus | 0.86 (0.52 to 0.99) | **3.76e-05** |
| Spearman’s r correlation coefficients, 95% confidence intervals (CIs), and p values are presented. | | |

| Supplementary Table 4: Comparison of soluble and insoluble poly(GP) levels among disease subgroups | | | | | | | | | | |
| --- | --- | --- | --- | --- | --- | --- | --- | --- | --- | --- |
|  |  | **ALS versus FTLD** | | | **ALS versus FTLD-MND** | | | **FTLD versus FTLD-MND** | | |
|  | **p value*** | **ALS** | **FTLD** | **p value** | **ALS** | **FTLD-MND** | **p value** | **FTLD** | **FTLD-MND** | **p value** |
| Cerebellum |  |  |  |  |  |  |  |  |  |  |
| Soluble Poly(GP) | **0.002** | 896  (817-1349) | 2679  (1158-3512) | **0.002** | 896  (817-1349) | 3220  (1453-4001) | **0.002** | 2679  (1158-3512) | 3220  (1453-4001) | 0.40 |
| Insoluble Poly(GP) | **0.0008** | 364  (266-473) | 982  (505-1247) | **0.0005** | 364  (266-473) | 896  (620-1129) | **0.0009** | 982  (505-1247) | 896  (620-1129) | 0.87 |
| Frontal Cortex |  |  |  |  |  |  |  |  |  |  |
| Soluble Poly(GP) | 0.10 | 330  (232-432) | 258  (191-320) | .. | 330  (232-432) | 378  (264-449) | .. | 258  (191-320) | 378  (264-449) | .. |
| Insoluble Poly(GP) | **0.003** | 255  (195-391) | 233  (128-291) | 0.36 | 255  (195-391) | 457  (291-601) | 0.04 | 233  (128-291) | 457  (291-601) | **0.001** |
| Motor Cortex |  |  |  |  |  |  |  |  |  |  |
| Soluble Poly(GP) | 0.37 | 322  (249-544) | 373  (273-566) | .. | 322  (249-544) | 406  (340-572) | .. | 373  (273-566) | 406  (340-572) | .. |
| Insoluble Poly(GP) | 0.34 | 208  (148-255) | 255  (179-335) | .. | 208  (148-255) | 252  (181-397) | .. | 255  (179-335) | 252  (181-397) | .. |
| Hippocampus |  |  |  |  |  |  |  |  |  |  |
| Soluble Poly(GP) | 0.56 | 256  (172-460) | 300  (215-381) | .. | 256  (172-460) | 355  (230-478) | .. | 300  (215-381) | 355  (230-478) | .. |
| Insoluble Poly(GP) | 0.85 | 122  (43-205) | 112  (80-147) | .. | 122  (43-205) | 135  (62-213) | .. | 112  (80-147) | 135  (62-213) | .. |
| Poly(GP) levels (ng/mg protein) presented as median (IQR). In total, associations with six variables were examined (i.e. disease subgroups, *C9ORF72* expansion size, *C9ORF72* variant 1, *C9ORF72* variant 3, age of onset, and survival after onset), and therefore p values lower than 0.0083 were considered significant after Bonferroni correction. Of note, only one of those variables is shown in this table (i.e. disease subgroups). *A Kruskal-Wallis rank sum test was performed to determine whether poly(GP) levels differed among disease subgroups (p<0.0083 considered significant after Bonferroni correction); when significant differences were detected a Wilcoxon rank sum test was used for pairwise comparisons (p<0.017 considered significant after Bonferroni correction). ALS=amyotrophic lateral sclerosis. FTLD=frontotemporal lobar degeneration. FTLD-MND=frontotemporal lobar degeneration with motor neuron disease. | | | | | | | | | | |

| Supplementary Table 5: Associations of soluble or insoluble poly(GP) levels with  *C9ORF72* expansion size, *C9ORF72* variants 1 and 3, and age at onset | | | |
| --- | --- | --- | --- |
|  | **Association** | **Spearman’s r (95% CI)** | **p value** |
| Cerebellum |  |  |  |
| Soluble poly(GP) | *C9ORF72* Expansion Size | -0.21 (-0.48 to 0.09) | 0.13 |
|  | *C9ORF72* Variant 1 | 0.24 (-0.04 to 0.50) | 0.09 |
|  | *C9ORF72* Variant 3 | 0.39 (0.11 to 0.63) | **0**.**005** |
|  | Age at Onset | 0.07 (-0.23 to 0.34) | 0.64 |
| Insoluble poly(GP) | *C9ORF72* Expansion Size | -0.05 (-0.34 to 0.23) | 0.71 |
|  | *C9ORF72* Variant 1 | 0.12 (-0.20 to 0.41) | 0.41 |
|  | *C9ORF72* Variant 3 | 0.35 (0.07 to 0.59) | 0.01 |
|  | Age at Onset | -0.02 (-0.32 to 0.28) | 0.91 |
| Frontal Cortex |  |  |  |
| Soluble poly(GP) | *C9ORF72* Expansion Size | -0.25 (-0.50 to 0.02) | 0.07 |
|  | *C9ORF72* Variant 1 | 0.15 (-0.16 to 0.44) | 0.30 |
|  | *C9ORF72* Variant 3 | 0.24 (-0.07 to 0.50) | 0.11 |
|  | Age at Onset | -0.21 (-0.48 to 0.09) | 0.14 |
| Insoluble poly(GP) | *C9ORF72* Expansion Size | -0.008 (-0.28 to 0.27) | 0.96 |
|  | *C9ORF72* Variant 1 | 0.11 (-0.22 to 0.41) | 0.47 |
|  | *C9ORF72* Variant 3 | 0.25 (-0.05 to 0.52) | 0.08 |
|  | Age at Onset | -0.28 (-0.55 to 0.003) | 0.04 |
| Spearman’s r correlation coefficients, 95% confidence intervals (CIs), and p values are presented. In total, associations with six variables were examined (i.e. disease subgroups, *C9ORF72* expansion size, *C9ORF72* variant 1, *C9ORF72* variant 3, age of onset, and survival after onset), and therefore p values lower than 0.0083 were considered significant after Bonferroni correction. Of note, only four of those variables are shown in this table. | | | |

| Supplementary Table 6: Associations of poly(GP) levels with survival after disease onset | | | |
| --- | --- | --- | --- |
|  | **Survival after onset** | **Hazard ratio (95% CI)** | **p value** |
| Cerebellum |  |  |  |
| Soluble poly(GP) | >Median | 0.93 (0.46 to 1.88) | 0.85 |
| Insoluble poly(GP) | >Median | 1.17 (0.59 to 2.31) | 0.65 |
| Total poly(GP) | >Median | 0.93 (0.46 to 1.88) | 0.85 |
| Frontal Cortex |  |  |  |
| Soluble poly(GP) | >Median | 0.88 (0.47 to 1.67) | 0.70 |
| Insoluble poly(GP) | >Median | 1.39 (0.73 to 2.65) | 0.32 |
| Total poly(GP) | >Median | 1.30 (0.69 to 2.42) | 0.42 |
| Data are hazard ratios with 95% confidence intervals (CI) or p value. In total, associations with six variables were examined (i.e. disease subgroups, *C9ORF72* expansion size, *C9ORF72* variant 1, *C9ORF72* variant 3, age of onset, and survival after onset), and therefore p values lower than 0.0083 were considered significant after Bonferroni correction. Of note, only one of those variables is shown in this table (i.e. survival after onset). A Cox proportional hazards regression model was used, using a dichotomous categorical variable with the median as the cut-off point. | | | |

| **Supplementary Table 7: Associations of soluble or insoluble poly(GP) levels with cognitive score or neuropathological diagnosis in the clinical cohort** | | | | | |
| --- | --- | --- | --- | --- | --- |
|  | **Cognitive score** | | **Neuropathological diagnosis**  **(ALS vs. FTLD-MND)** | | |
|  | **Spearman’s r (95% CI)** | **p value** | **ALS** | **FTLD-MND** | **p value** |
| **Cerebellum** |  |  |  |  |  |
| Soluble poly(GP) | 0.70 (0.27 to 0.89) | **0.005** | 830 (788-1282) | 2920 (2016-3269) | **0.003** |
| Insoluble poly(GP) | 0.74 (0.36 to 0.91) | **0.002** | 321 (176-427) | 845 (597-976) | **0.003** |
| **Frontal cortex** |  |  |  |  |  |
| Soluble poly(GP) | 0.09 (-0.45 to 0.59) | 0.74 | 313 (256-410) | 391 (371-405) | 0.39 |
| Insoluble poly(GP) | -0.08 (-0.58 to 0.47) | 0.71 | 265 (204-386) | 374 (283-456) | 0.27 |
| **Motor cortex** |  |  |  |  |  |
| Soluble poly(GP) | 0.28 (-0.29 to 0.70) | 0.31 | 310 (241-402) | 458 (350-590) | 0.07 |
| Insoluble poly(GP) | 0.10 (-0.45 to 0.59) | 0.72 | 223 (151-272) | 328 (270-418) | 0.09 |
| **Hippocampus** |  |  |  |  |  |
| Soluble poly(GP) | 0.17 (-0.39 to 0.64) | 0.53 | 217 (176-327) | 398 (381-517) | 0.04 |
| Insoluble poly(GP) | -0.00 (-0.53 to 0.52) | 0.91 | 117 (45-145) | 160 (133-264) | 0.22 |
| Spearman’s r correlation coefficients, 95% confidence intervals (CIs), median (IQR), and p values are presented. Given the assessment of poly(GP) levels (ng/mg) with two variables (i.e. cognitive score and neuropathological diagnosis), p values lower than 0.025 were considered significant after Bonferroni correction. ALS=amyotrophic lateral sclerosis. FTLD-MND=frontotemporal lobar degeneration with motor neuron disease. | | | | | |

| **Supplementary Table 8: Associations of poly(GP) levels with pTDP-43 pathology in the clinical cohort** | | | | |
| --- | --- | --- | --- | --- |
|  | **pTDP-43 in Frontal Cortex** | | **pTDP-43 in Hippocampus** | |
|  | **Spearman’s r (95% CI)** | **p value** | **Spearman’s r (95% CI)** | **p value** |
| **Cerebellum** |  |  |  |  |
| Soluble poly(GP) | 0.76 (0.35 to 0.93) | **0.002** | 0.67 (0.18 to 0.87) | **0.006** |
| Insoluble poly(GP) | 0.59 (0.07 to 0.87) | **0.02** | 0.60 (0.12 to 0.85) | **0.02** |
| Total poly(GP) | 0.69 (0.22 to 0.92) | **0.005** | 0.69 (0.25 to 0.89) | **0.005** |
| **Frontal cortex** |  |  |  |  |
| Soluble poly(GP) | 0.08 (-0.51 to 0.60) | 0.78 | 0.36 (-0.17 to 0.82) | 0.19 |
| Insoluble poly(GP) | -0.04 (-0.67 to 0.56) | 0.90 | 0.04 (-0.56 to 0.61) | 0.87 |
| Total poly(GP) | 0.05 (-0.61 to 0.60) | 0.87 | 0.12 (-0.41 to 0.62) | 0.67 |
| **Motor cortex** |  |  |  |  |
| Soluble poly(GP) | 0.36 (-0.25 to 0.81) | 0.19 | 0.48 (-0.02 to 0.83) | 0.07 |
| Insoluble poly(GP) | 0.18 (-0.38 to 0.68) | 0.52 | 0.20 (-0.35 to 0.64) | 0.46 |
| Total poly(GP) | 0.24 (-0.31 to 0.74) | 0.38 | 0.38 (-0.15 to 0.77) | 0.16 |
| **Hippocampus** |  |  |  |  |
| Soluble poly(GP) | 0.43 (-0.14 to 0.82) | 0.11 | 0.22 (-0.35 to 0.71) | 0.43 |
| Insoluble poly(GP) | 0.33 (-0.24 to 0.75) | 0.23 | 0.17 (-0.40 to 0.66) | 0.53 |
| Total poly(GP) | 0.41 (-0.16 to 0.81) | 0.13 | 0.21 (-0.32 to 0.69) | 0.46 |
| Spearman’s r correlation coefficients and 95% confidence intervals (CIs). P values lower than 0.025 were considered significant after Bonferroni correction. | | | | |

| Supplementary Table 9: Associations between soluble and insoluble poly(GA) levels in the cerebellum | | |
| --- | --- | --- |
|  | **Spearman’s r (95% CI)** | **p value** |
| Soluble vs. insoluble poly(GA) in the full cohort (n=55) | 0.47 (0.23 to 0.65) | **0.0003** |
| Soluble vs. insoluble poly(GA) in the clinical cohort (n=15) | 0.65 (0.17 to 0.92) | **0.01** |
| Spearman’s r correlation coefficients, 95% confidence intervals (CIs), and p values are presented. | | |

| Supplementary Table 10: Comparison of cerebellar poly(GA) levels between disease subgroups | | | | | | | | | | |
| --- | --- | --- | --- | --- | --- | --- | --- | --- | --- | --- |
|  |  | **ALS versus FTLD** | | | **ALS versus FTLD-MND** | | | **FTLD versus FTLD-MND** | | |
|  | **p value*** | **ALS** | **FTLD** | **p value** | **ALS** | **FTLD-MND** | **p value** | **FTLD** | **FTLD-MND** | **p value** |
| Cerebellum |  |  |  |  |  |  |  |  |  |  |
| Soluble Poly(GA) | 0.04 | 4001  (3794-4469) | 4629  (4154-5409) | .. | 4001  (3794-4469) | 4723  (4385-5272) | .. | 4629  (4154-5409) | 4723  (4385-5272) | .. |
| Insoluble Poly(GA) | 0.02 | 4930  (4356-6507) | 7066  (6274-9833) | .. | 4930  (4356-6507) | 6804  (6310-7772) | .. | 7066  (6274-9833) | 6804  (6310-7772) | .. |
| Total Poly(GA) | 0.009 | 8877  (8327-10616) | 11723  (10666-15054) | .. | 8877  (8327-10616) | 11677  (10627-13673) | .. | 11723  (10666-15054) | 11677  (10627-13673) | .. |
| Poly(GA) levels (ng/mg protein) presented as median (IQR). In total, associations with six variables were examined (i.e. disease subgroups, *C9ORF72* expansion size, *C9ORF72* variant 1, *C9ORF72* variant 3, age of onset, and survival after onset), and therefore p values lower than 0.0083 were considered significant after Bonferroni correction. Of note, only one of those variables is shown in this table (i.e. disease subgroups). *A Kruskal-Wallis rank sum test was performed to determine whether poly(GA) levels differed among disease subgroups (p<0.0083 considered significant after Bonferroni correction). Since no significant differences were detected among subgroups (p>0.0083), pairwise comparisons were not analyzed. ALS=amyotrophic lateral sclerosis. FTLD=frontotemporal lobar degeneration. FTLD-MND=frontotemporal lobar degeneration with motor neuron disease. | | | | | | | | | | |

| Supplementary Table 11: Associations of cerebellar poly(GA) levels with *C9ORF72* expansion size, *C9ORF72* variants 1 and 3, and age at onset | | | |
| --- | --- | --- | --- |
|  | **Association** | **Spearman’s r (95% CI)** | **p value** |
| Cerebellum |  |  |  |
| Soluble poly(GA) | *C9ORF72* Expansion Size | -0.12 (-0.38 to 0.14) | 0.39 |
|  | *C9ORF72* Variant 1 | 0.12 (-0.19 to 0.40) | 0.42 |
|  | *C9ORF72* Variant 3 | 0.28 (-0.01 to 0.52) | 0.05 |
|  | Age at Onset | 0.30 (0.03 to 0.54) | 0.03 |
| Insoluble poly(GA) | *C9ORF72* Expansion Size | -0.26 (-0.51 to 0.01) | 0.06 |
|  | *C9ORF72* Variant 1 | 0.30 (0.008 to 0.54) | 0.04 |
|  | *C9ORF72* Variant 3 | 0.45 (0.22 to 0.65) | **0.001** |
|  | Age at Onset | 0.16 (-0.14 to 0.43) | 0.24 |
| Total poly(GA) | *C9ORF72* Expansion Size | -0.24 (-0.50 to 0.04) | 0.08 |
|  | *C9ORF72* Variant 1 | 0.25 (-0.04 to 0.51) | 0.08 |
|  | *C9ORF72* Variant 3 | 0.46 (0.22 to 0.66) | **0.0009** |
|  | Age at Onset | 0.17 (-0.13 to 0.44) | 0.23 |
| Spearman’s r correlation coefficients, 95% confidence intervals (CIs), and p values are presented. In total, associations with six variables were examined (i.e. disease subgroups, *C9ORF72* expansion size, *C9ORF72* variant 1, *C9ORF72* variant 3, age of onset, and survival after onset), and therefore p values lower than 0.0083 were considered significant after Bonferroni correction. Of note, only four of those variables are shown in this table. | | | |

| Supplementary Table 12: Associations of cerebellar poly(GA) levels with survival after disease onset | | | |
| --- | --- | --- | --- |
|  | **Survival after onset** | **Hazard ratio (95% CI)** | **p value** |
| Cerebellum |  |  |  |
| Soluble poly(GA) | >Median | 0.85 (0.43 to 1.66) | 0.63 |
| Insoluble poly(GA) | >Median | 0.73 (0.41 to 1.30) | 0.29 |
| Total poly(GA) | >Median | 0.75 (0.41 to 1.41) | 0.38 |
| Data are hazard ratios with 95% confidence intervals (CI) or p value. In total, associations with six variables were examined (i.e. disease subgroups, *C9ORF72* expansion size, *C9ORF72* variant 1, *C9ORF72* variant 3, age of onset, and survival after onset), and therefore p values lower than 0.0083 were considered significant after Bonferroni correction. Of note, only one of those variables is shown in this table (i.e. survival after onset). A Cox proportional hazards regression model was used, using a dichotomous categorical variable with the median as the cut-off point. | | | |

| **Supplementary Table 13: Associations of cerebellar poly(GA) levels with cognitive score or neuropathological diagnosis in the clinical cohort** | | | | | |
| --- | --- | --- | --- | --- | --- |
|  | **Cognitive score** | | **Neuropathological diagnosis**  **(ALS vs. FTLD-MND)** | | |
|  | **Spearman’s r (95% CI)** | **p value** | **ALS** | **FTLD-MND** | **p value** |
| **Cerebellum** |  |  |  |  |  |
| Soluble poly(GA) | 0.16  (-0.38 to 0.68) | 0.57 | 4058  (3796-4426) | 5102  (4757-5448) | **0.016** |
| Insoluble poly(GA) | 0.43  (-0.19 to 0.89) | 0.11 | 5132  (4568-6953) | 7089  (5837-7605) | 0.14 |
| Total poly(GA) | 0.35  (-0.29 to 0.85) | 0.20 | 8918  (8625-10783) | 11963  (11262-12877) | 0.07 |
| Spearman’s r correlation coefficients, 95% confidence intervals (CIs), median (IQR), and p values are presented. Given the assessment of poly(GA) levels with two variables (i.e. cognitive score and neuropathological diagnosis), p values lower than 0.025 were considered significant after Bonferroni correction. ALS=amyotrophic lateral sclerosis. FTLD-MND=frontotemporal lobar degeneration with motor neuron disease. | | | | | |

| **Supplementary Table 14: Associations of poly(GA) levels with pTDP-43 pathology in the clinical cohort** | | | | |
| --- | --- | --- | --- | --- |
|  | **pTDP-43 in Frontal Cortex** | | **pTDP-43 in Hippocampus** | |
|  | **Spearman’s r (95% CI)** | **p value** | **Spearman’s r (95% CI)** | **p value** |
| **Cerebellum** |  |  |  |  |
| Soluble poly(GA) | 0.72 (0.31 to 0.90) | **0.004** | 0.64 (0.23 to 0.83) | **0.01** |
| Insoluble poly(GA) | 0.73 (0.26 to 0.94) | **0.003** | 0.57 (-0.05 to 0.90) | 0.03 |
| Total poly(GA) | 0.78 (0.33 to 0.99) | **0.001** | 0.64 (0.11 to 0.92) | **0.01** |
| Spearman’s r correlation coefficients and 95% confidence intervals (CIs). P values lower than 0.025 were considered significant after Bonferroni correction. | | | | |

**Supplementary Figure**

**Supplementary Fig. 1: Validation of poly(GA) MSD sandwich immunoassay**

(**a**) To validate specificity of the poly(GA) assay, synthetic peptides representing each possible c9RAN protein translated from sense or antisense transcripts of the expanded *C9ORF72* repeat were diluted in Tris-buffered saline (TBS) and assayed (1 µg per well in duplicate wells). Response values correspond to intensity of emitted light upon electrochemical stimulation of the assay plate using the MSD QUICKPLEX SQ120, from which the background response in wells containing only TBS was subtracted. Data presented as mean + standard error of mean of responses in duplicate wells. (**b**) Poly(GA) immunoassay response values in soluble cerebellar homogenates from four ALS patients and four c9ALS patients. The median in a given group is denoted by a solid horizontal line.
